# Dimensionality reduction of neuronal degeneracy reveals two interfering physiological mechanisms

**DOI:** 10.1093/pnasnexus/pgae415

**Published:** 2024-09-19

**Authors:** Arthur Fyon, Alessio Franci, Pierre Sacré, Guillaume Drion

**Affiliations:** Department of Electrical Engineering and Computer Science, University of Liège, Liège B-4000, Belgium; Department of Electrical Engineering and Computer Science, University of Liège, Liège B-4000, Belgium; WEL-T Department, WEL Research Institute, Wavre B-1300, Belgium; Department of Electrical Engineering and Computer Science, University of Liège, Liège B-4000, Belgium; Department of Electrical Engineering and Computer Science, University of Liège, Liège B-4000, Belgium

**Keywords:** ion channel correlation, neuronal variability, neuronal degeneracy, conductance-based model, neuromodulation

## Abstract

Neuronal systems maintain stable functions despite large variability in their physiological components. Ion channel expression, in particular, is highly variable in neurons exhibiting similar electrophysiological phenotypes, which raises questions regarding how specific ion channel subsets reliably shape intrinsic properties of neurons. Here, we use detailed conductance-based modeling to explore how stable neuronal function is achieved despite variability in channel composition among neurons. Using dimensionality reduction, we uncover two principal dimensions in the channel conductance space that capture most of the variance of the observed variability. These two dimensions correspond to two sources of variability that originate from distinct physiologically relevant mechanisms underlying the regulation of neuronal activity, providing quantitative insights into how channel composition is linked to the electrophysiological activity of neurons. These insights allow us to understand and design a model-independent, reliable neuromodulation rule for variable neuronal populations.

**Significance Statement**

Neuronal electrical activity is primarily regulated by a variety of transmembrane proteins known as ion channels. These channels exhibit substantial intraindividual variability in their number but neurons nonetheless maintain their proper function under physiological conditions—a concept known as degeneracy. This article is intended to deepen our understanding of how different ion channels interact to regulate neuronal function. Specifically, we use dimensionality reduction techniques and computational neuron models to demonstrate that the distribution of ion channels arises from two distinct physiological mechanisms. Studying the interaction between these two mechanisms sheds light on how the expression levels of different ion channels are linked to each other and determine neuronal activity. Such insights could significantly enhance the design of electrophysiological experiments.

## Introduction

A remarkable property of nervous systems is their ability to maintain stable functions despite large variability and turnover of the underlying physiological components. This observation has led to the understanding that neuron electrophysiological properties are shaped by the coordinated expression of potentially large subsets of ion channels (1), which represent a substantial challenge in any attempt to link ion channel properties with neuron electrophysiological signature.

In recent decades, a combination of experimental and computational work has provided insights into the relationship between the densities of ion channels and neuronal signaling. First, it has been clarified that different combinations of ion channels can lead to similar activity despite substantial variation in channel densities (2–6), as a result of functional overlap in channel voltage- and time-dependent properties (1, 7). Second, it has been shown experimentally that ion channel expression correlates positively in the same neuron type, while the correlations vary among different neuron types (8–13). It has been revealed that these positive correlations in ion channel expression emerge from physiologically plausible homeostatic rules (14). One could thus argue that specific correlations in channel expression are an important neuronal signature. Third, consistent neuromodulatory effects despite the large variability of ion channel expression (also called reliable neuromodulation) has been shown to often occur through a concomitant action on several channel subtypes (8, 15–18), which highlights the importance of understanding the mechanisms that link the density of ion channels and neuronal signaling.

Although this body of work has deepened our understanding of how ion channels shape neuronal activity, many important questions remain. First, although most studies have reported positive correlations in channel gene mRNA expression, studies on correlations in actual conductance values have revealed a less clear picture. Correlations in conductance values are observed, but the correlation coefficient can vary and it can be either positive or negative depending on the ion channel subtype and neuron subtype (19–22). In addition, correlations in both channel gene mRNA expression and conductance values can be dependent on activity and neuromodulation (23, 24). Given these negative correlations in conductance values, the question arises of what potentially complex mechanism might link channel gene mRNA and protein expressions. Here, we attempt to answer this question by analyzing how positive and negative conductance correlations arise in highly degenerate parameter sets of two different conductance-based models. We show that pairwise correlations in channel conductance are the result of two interfering mechanisms. Such interference is activity-dependent, which results in activity-dependent correlation levels. Another unanswered question involves the fact that, at present, our understanding of how ion channels shape neuronal activity remains largely qualitative. The lack of a concrete mechanistic understanding makes it extremely difficult to quantify how specific changes in ion channel density affect neuronal output, which in turn makes the study of reliable neuromodulation laborious. Here, we provide such a mechanistic understanding through a dimensionality reduction analysis of the two degenerate parameter sets. The geometry of the principal components (PCs) found by dimensionality reduction methods is fully explained by the geometry of the sensitive directions in the maximal conductance space, as revealed by using feedback control ideas (7). This analysis permits the derivation of a simple, physiologically plausible rule explaining how neuromodulation can be achieved reliably in highly degenerate neurons.

## Results

### Neuronal degeneracy in conductance-based models is associated with variable pairwise correlations in channel conductances

**Fig. 1.**
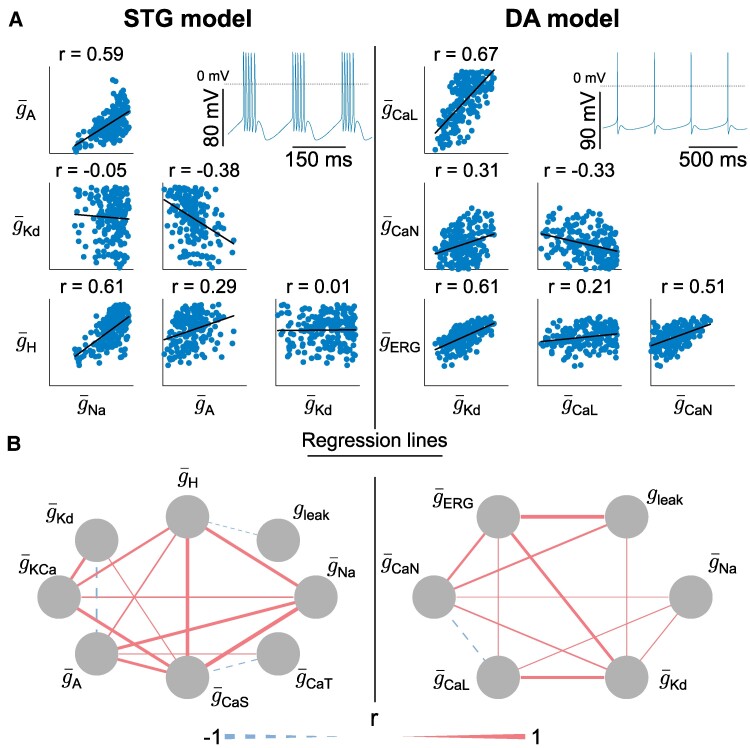
Neuronal degeneracy in conductance-based models is associated with variable pairwise correlations in channel conductances. A) Scatter plot matrices of random sampling populations in the conductance spaces for the STG model (left) and the DA model (right), along with regression lines. The pairs depicted here do not represent all conductances of the models and are randomly chosen to illustrate the variable correlations, expressed by the Pearson correlation coefficient (*r*). All conductances are expressed in mS/cm^2^. The bottom left corner of each scatter plot represents the origin of the conductance space. For the STG model, ${\bar{g}}_{A}$ ranges up to 600, ${\bar{g}}_{\mathrm{Kd}}$ to 350, ${\bar{g}}_{H}$ to 0.7, and ${\bar{g}}_{\mathrm{Na}}$ to 8,000. For the DA model, ${\bar{g}}_{\mathrm{CaL}}$ ranges up to 0.1, ${\bar{g}}_{\mathrm{CaN}}$ to 0.12, ${\bar{g}}_{\mathrm{ERG}}$ to 0.25, and ${\bar{g}}_{\mathrm{Kd}}$ to 20. The dotted line on the voltage traces corresponds to 0mV. B) Correlation graphs of all conductances of the random sampling populations for the STG model (left) and DA model (right). A dashed (solid) line indicates a negative (positive) pairwise correlation. The thickness of the line represents the absolute value of the correlation. Correlations below a certain threshold, corresponding to the inverse of the number of conductances in the considered model (one-eighth and one-sixth in the STG and DA models respectively), are not shown.

We initially created variable sets of conductances leading to stable firing patterns in two different neuron conductance-based models (Fig. 1): a stomatogastric (STG) neuron model (25) (left) and a dopaminergic (DA) neuron model (adapted from (26)) (right). All simulations and analyses were performed on these two different models to avoid uncovering model-specific features, but rather to focus on general properties. Each parameter set was created through random sampling followed by a post-processing procedure that selected models sharing specific firing pattern characteristics (4). Each model was studied in its nominal firing pattern: burst firing for the STG neuron model, and slow tonic spiking for the DA neuron model (see Materials and methods). An example of each firing pattern is shown at the top, right of each panel in Fig. 1A.

Figure 1A shows a scatter plot matrix of ion channel maximal conductances for a subset of ion channel types in both models, as well as the correlation computed for each pair. As observed in previous experimental and computational work (1, 20), correlations can vary markedly between different pairs of conductances, from strongly positive (such as ${\bar{g}}_{\mathrm{Na}}$ and ${\bar{g}}_{A}$ in STG model), to negative (such as ${\bar{g}}_{A}$ and ${\bar{g}}_{\mathrm{Kd}}$ in STG model), or seemingly uncorrelated. This highlights the strong degeneracy of both conductance-based models, despite the fact that they maintain their respective firing activity using different types of ion channels.

To gain deeper insights into how conductances correlate to maintain robust firing activity, we represent the pairwise correlations between all conductances using correlation graphs (Fig. 1B). Each node represents a conductance, the thickness of the edges connecting each node represents the strength of the correlation, and the color of each edge represents the correlation sign (red for positive and blue for negative). These two graphs show similar trends for the two models: correlations between ion channels are mostly positive, but there are also negative correlations in a small subset of conductance pairs. This is intriguing for two reasons.

First, to maintain similar firing activity, one would expect conductances that are sources of currents of the same sign to correlate negatively, whereas conductances that are sources of currents of the opposite sign would correlate positively. This would allow the global transmembrane current, hence excitability, to be maintained at a steady level. However, this is not what is observed in Fig. 1B. If we take the example of ${\bar{g}}_{\mathrm{CaS}}$ in the STG model, which is a source of inward current, it can correlate either negatively or positively with other sources of inward currents (${\bar{g}}_{\mathrm{CaT}}$ and ${\bar{g}}_{\mathrm{Na}}$, respectively). Likewise, outward current sources can correlate either negatively or positively with other outward sources (i.e. ${\bar{g}}_{\mathrm{Kd}}$ with ${\bar{g}}_{A}$ and ${\bar{g}}_{\mathrm{KCa}}$ in the STG model). The same observation can be made for the DA neuron model.

Second, experimental studies on the correlation between ion channel mRNA and computational models of neuronal homeostasis have uncovered the existence and emergence of neuron-dependent, strictly positive correlations in channel densities (1, 13, 27). A similar trend emerges from our dataset, where the majority of correlations are indeed positive. However, negative correlations are also observed in our dataset, as well as experimental data on ion channel conductances (20). This suggests that correlations emerging from homeostatic rules are important for the maintenance of robust firing activity, but that some other mechanisms must be at play.

### A few PCs capture neuronal degeneracy but do not single out channel functions

**Fig. 2.**
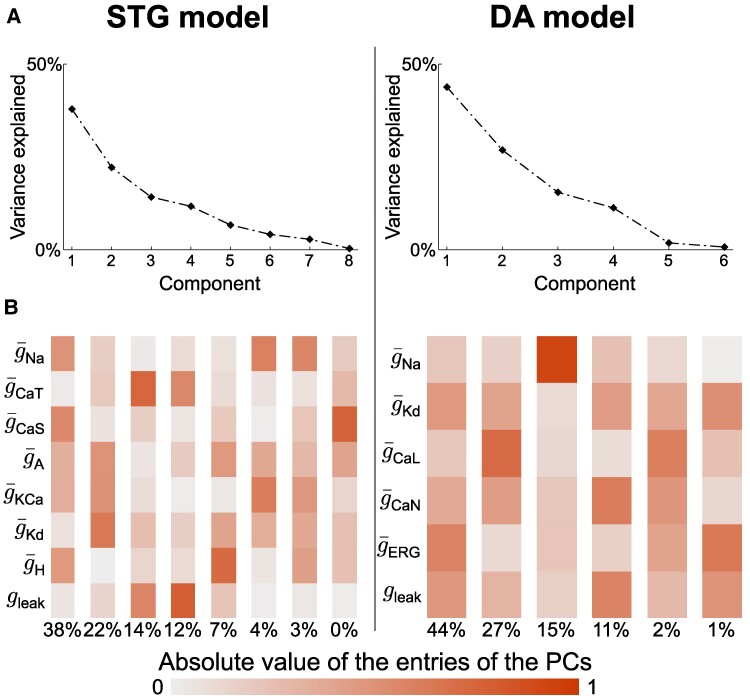
A few PCs capture neuronal degeneracy but do not single out channel functions. A) Screen plot of PCA applied to the conductance spaces of random sampling populations for the STG model (left) and the DA model (right). B) Absolute values of the entries of the PCs in the conductance space for the STG model (left) and the DA model (right).

As pairwise correlations between conductances alone did not provide much insight into how ion channels correlate to maintain robust firing activity, we performed principal component analysis (PCA) of both random sampling sets in an attempt to uncover low-dimensional subspaces in the data. We observed that a limited number of PCs, namely, four for the STG model and three for the DA model, accounts for more than 80% of the total variances in the data (Fig. 2A). We chose to focus our analysis on these significant PCs. The first PC accounted for approximately 40% of the variance in both models. This observation is encouraging, as it shows that the mechanisms that drive conductance joint distribution in neuron models are low-dimensional, which is key for interpretability.

We then extracted the contribution of each conductance in each of the PCs, with the hope of observing a pattern that would allow us to make predictions on the biophysics behind these components (Fig. 2B). However, the results were difficult to interpret, as a variety of conductances contributed to the different PCs for both models. Moreover, conductances that made substantial contributions to the first PC in one model did not do so in the other (e.g. see the role of ${\bar{g}}_{\mathrm{Na}}$ or $g_{\mathrm{leak}}$ in the two models), which prevented the extraction of a model-independent rule from a naive analysis focusing on the role of single conductances. Although this last observation might seem unsurprising, as both models relate to different neurons exhibiting different firing patterns from different ion channels, we still aim to find some common, general mechanisms that might rule the degeneracy in ion channel conductances.

### Dominant PC captures homogeneous scaling of maximal conductances

**Fig. 3.**
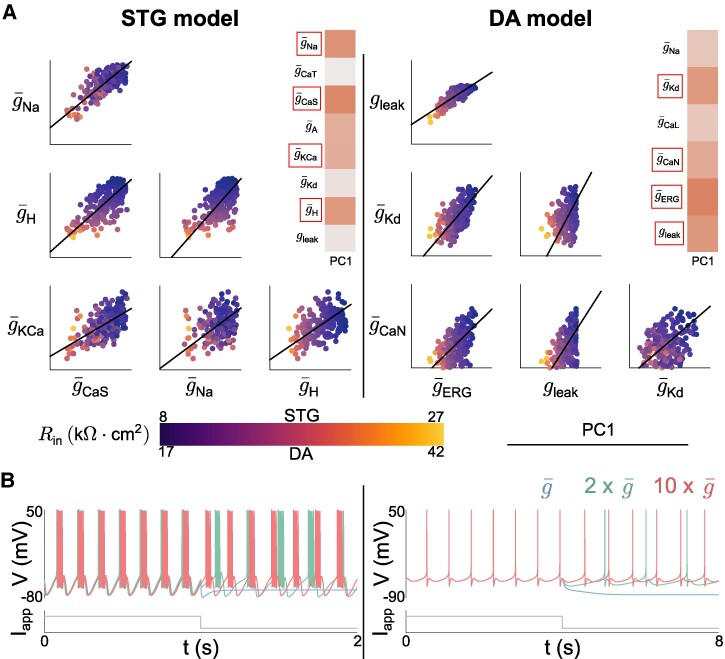
Dominant PC captures homogeneous scaling of maximal conductances. A) Scatter plot matrices of random sampling populations in the conductance spaces for the STG model (left) and the DA model (right) along with the direction of PC1, color coded based on the input resistance. The scatter plots shown are associated with the conductances having the largest entries (in absolute value) in the first PC. All conductances are expressed in mS/cm^2. The bottom left corner of each 2D subspace represents the origin of the conductance space. For the STG model, ${\bar{g}}_{\mathrm{Na}}$ ranges up to 8,000, ${\bar{g}}_{H}$ to 0.7, ${\bar{g}}_{\mathrm{KCa}}$ to 250 and ${\bar{g}}_{\mathrm{CaS}}$ to 50. For the DA model, $g_{\mathrm{leak}}$ ranges up to 0.02, ${\bar{g}}_{\mathrm{Kd}}$ to 20, ${\bar{g}}_{\mathrm{CaN}}$ to 0.12 and ${\bar{g}}_{\mathrm{ERG}}$ to 0.25. B) Simulations illustrating the effect of homogeneous scaling for the STG model (left) and the DA model (right). A random model from the scatter plot in (A) receives an inhibitory input. The same experiment is then conducted with all conductances multiplied by 2 and 10.

As the first principal component (PC1) accounted for a large portion of the variability in the data for both models (approximately 40%), we further analyzed its role by creating scatter plots of conductance values for a subset of four conductances that play dominant roles in PC1 (Fig. 3A). Interestingly, according to these scatter plots, all conductances that play significant roles in PC1 are strongly positively correlated with each other in both models. This is highly reminiscent of previous observations in channel mRNA data or the channel correlations emerging from models of neuronal homeostasis (14, 27, 28). In particular, such positive correlations follow a direction passing roughly through the origin.

This direction is close to the homogeneous scaling direction in the maximal conductances. The direction of homogeneous scaling corresponds to the total least squares regression direction without intercept, i.e. to the direction connecting the origin of the conductance space to the center of mass of the degeneracy set. This center of mass represents the means of every type of conductance across the population. While pairwise homogeneous scaling is only evident in a subset of ion channels, this observation extends to the entire conductance space. The alignment between PC1 and homogeneous scaling in the full conductance space was robustly confirmed in both the STG and the DA models, with a notable 0.8 alignment in the former and a remarkable 0.9 alignment in the latter. This alignment was computed as the dot product between the unit vectors along PC1 and homogeneous scaling direction. Alternatively, it can be interpreted as the cosine of the angle formed by these two directions in the high-dimensional space of conductances.

The dominant role of homogeneous scaling of conductances in neuronal degeneracy can be understood by its functional significance. Such homogeneous scaling can emerge from homeostatic models of ion channel expression, where the slope between a pair of conductances correlates with the type of neuronal activity (14). This slope is determined by the ratio of regulation time constants. Homogeneous scaling also permits modulation of the neuron response to external inputs while its intrinsic firing pattern is maintained unaffected. Indeed, increasing all conductances by a common factor permits an increase in the global membrane permeability, which decreases the responsiveness to external input through a decrease in input resistance $R_{\mathrm{in}}$ (Fig. 3B). As indicated by the color coding in Fig. 3A, the direction of PC1, which represents homogeneous scaling, aligns with the variability in neuron input resistance in both models. At the same time, it does not affect the ratio between channel conductances, thus maintaining firing activity. Therefore, homogeneous scaling plays a critical role in excitability modulation and homeostasis.

### Normalization of the datasets by the input resistance reveals that the secondary PCs capture degenerate conductance ratios

Analysis of the remaining meaningful principal components (PC2, PC3, and PC4 in the STG model, and PC2 and PC3 in the DA model) should shed light on the physiological origin of most of the remaining variance in the data. However, these PCs have highly variable slopes in the different conductance planes, making the analysis less straightforward than for PC1. The effect of homogeneous scaling is intertwined with the other potential origins of degeneracy in neuron model populations, which complicates matters further.

**Fig. 4.**
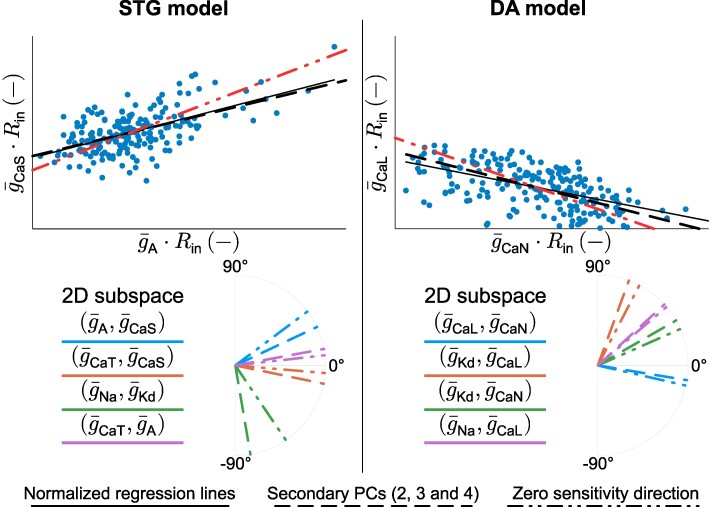
Normalization of the dataset by the input resistance reveals that the secondary PCs capture degenerate conductance ratios that maintain neuronal activity. Scatter plots (top) of random sampling populations in the $\left({\bar{g}}_{A},{\bar{g}}_{\mathrm{CaL}}\right)$ 2D subspace for the STG model (left) and the $\left({\bar{g}}_{\mathrm{CaL}},{\bar{g}}_{\mathrm{CaN}}\right)$ 2D subspace for the DA model (right), normalized by the input resistance, along with the regression lines of the normalized dataset (solid line), secondary PCs of the non-normalized dataset (dashed line), and the zero-sensitivity direction (dash-dotted line). The bottom left corners of the scatter plots represent the origin of the conductance space, and the ranges are irrelevant. Polar plots of secondary PCs and the zero sensitivity direction in randomly chosen 2D subspaces of the conductance space (bottom).

To circumvent this problem, we removed the effect of PC1 by normalizing the dataset by neuron input resistance, thereby eliminating the effect of homogeneous scaling. This was achieved by multiplying each conductance by $R_{\mathrm{in}}$ (i.e. dividing by the input conductance $g_{\mathrm{in}}$). The regression lines of the normalized dataset almost perfectly coincide with the secondary PCs of the non-normalized dataset (Fig. 4), demonstrating that normalization by input resistance effectively suppresses the effect of PC1.

Once the effect of homogeneous scaling is removed, the remaining variability corresponds to changes in conductance ratios that do not impact neuron input resistance. Degeneracy in conductance ratios can be quantified by leveraging the concept of dynamic input conductances (DICs) (7), which provides a way of linking channel conductance ratios with firing activity. In short, it was shown that the dynamical effects of ion channel gating on neuron activity could be captured by a few voltage-dependent conductance curves (DIC) acting on separate timescales. For a bursting neuron, three timescales are sufficient: a fast timescale characterizing spike upstroke; a slow timescale characterizing spike downstroke, neuron excitability type and rest-spike bistability; and an ultraslow timescale characterizing burst parameters such as period and duty cycle. The value of each DIC at the threshold potential on each timescale determines firing activity, and each parameter set leading to similar DIC values leads to similar firing activities. We exploited this last property to understand the variability that remains in the normalized dataset by identifying directions of zero sensitivity in the maximal conductance space, i.e. directions along which changes in maximal conductances do not affect DIC values at threshold, and hence lead to similar spiking behavior (7).

We then verified if variability in conductance ratio leading to similar DIC values was the dominant source of degeneracy in the normalized dataset by computing zero-sensitivity directions of the slow DIC in both STG and DA neuron models and comparing these directions with the secondary PCs of the original dataset (PC2, PC3, and PC4). Indeed, we found that the effect of the slow DIC was dominant on degeneracy, as the slow DIC is the main player in determining neural excitability types by governing spiking-to-bursting transitions and the regulation of cellular rest-spike bistability (for further details, see Materials and methods). In both models, the zero-sensitivity directions strongly align with one of the secondary PCs in the original random sampling set (Fig. 4), and thus with the regression line of the normalized dataset. This confirmed that the second origin of degeneracy in ion channel expression can be explained by the existence of degenerate conductance ratios that create similar membrane dynamical properties.

Degeneracy in conductance ratios is also functionally significant for robust neuronal signaling. Relying on different conductance ratios to create similar firing activity allows the creation of heterogeneity in response to external perturbations such as changes in temperature or pH (29, 30), as well as specific ion channel blockades or dysfunction, which increases neuronal robustness. It also creates variable responses to exogenous neuroactive drugs and allows for compensation during long-lasting drug exposure or a genetic defect in the expression of a specific channel.

### An alternative approach to building degenerate parameter sets allows the effect of homogeneous scaling to be separated from variability in conductance ratios

To better understand how homogeneous scaling and variability in conductance ratios interfere with each other, we constructed a new dataset that allowed us to separate these two effects. We created datasets of similar firing patterns by allowing randomness in all conductances but one per timescale, and adapting the remaining conductances to ensure that DIC values are kept constant (for further details, see Materials and methods). Importantly, to be able to separate the effect of homogeneous scaling from other sources of ion channel degeneracy, we normalized DIC values by $g_{\mathrm{leak}}$. This normalization allows the creation of variable conductance ratios that barely affects homogeneous scaling, which is itself mostly captured through variability in $g_{\mathrm{leak}}$. We decided to perform normalization using $g_{\mathrm{leak}}$ instead of $R_{\mathrm{in}}$ for computational efficiency, as $R_{\mathrm{in}}$ depends on all conductances and is voltage-dependent. Note that homogeneous scaling is equally well captured using $R_{\mathrm{in}}$ or $g_{\mathrm{leak}}$, since the leak conductance is the dominant current source below the threshold potential in both models.

**Fig. 5.**
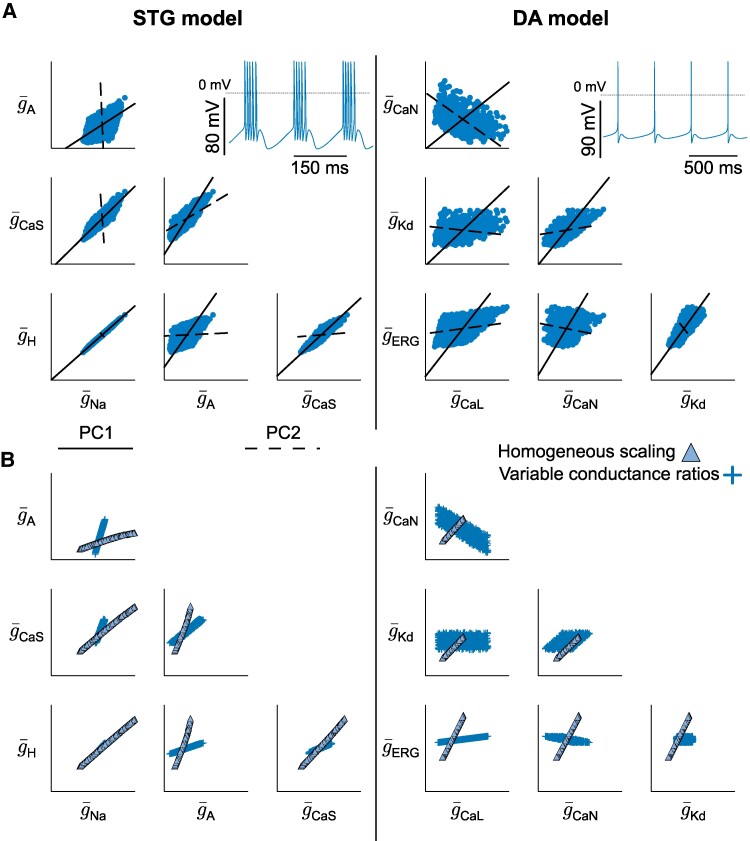
An alternative approach to building degenerate parameter sets allows the effect of homogeneous scaling to be separated from variability in conductance ratios. A) Scatter matrices of custom generated populations in the conductance spaces for the STG model (left) and the DA model (right) along with the directions of PC1 and PC2. The 2D subspaces shown here do not represent all conductances of the models and are randomly chosen. All conductances are expressed in mS/cm^2. The bottom left corner of each 2D subspace represents the origin of the conductance space. For the STG model, ${\bar{g}}_{A}$ ranges up to 600, ${\bar{g}}_{\mathrm{CaS}}$ to 50, ${\bar{g}}_{H}$ to 0.7 and ${\bar{g}}_{\mathrm{Na}}$ to 8,000. For the DA model, ${\bar{g}}_{\mathrm{CaN}}$ ranges up to 0.12, ${\bar{g}}_{\mathrm{Kd}}$ to 20, ${\bar{g}}_{\mathrm{ERG}}$ to 0.25 and ${\bar{g}}_{\mathrm{CaL}}$ to 0.1. The dotted line on the voltage traces corresponds to the 0mV line. B) Scatter matrices of custom generated populations in the conductance spaces for the STG model (left) and the DA model (right), isolating the effects of homogeneous scaling only (resp. variability in conductance ratios) shown as triangles (resp. crosses). The 2D subspaces are the same as in a). All conductances are expressed in mS/cm^2. The bottom left corner of each 2D subspace represents the origin of the conductance space. For the STG model, ${\bar{g}}_{A}$ ranges up to 600, ${\bar{g}}_{\mathrm{CaS}}$ to 50, ${\bar{g}}_{H}$ to 0.7 and ${\bar{g}}_{\mathrm{Na}}$ to 8,000. For the DA model, ${\bar{g}}_{\mathrm{CaN}}$ ranges up to 0.12, ${\bar{g}}_{\mathrm{Kd}}$ to 20, ${\bar{g}}_{\mathrm{ERG}}$ to 0.25 and ${\bar{g}}_{\mathrm{CaL}}$ to 0.1.

The dataset constructed using this approach created neurons exhibiting similar firing activities (see Fig. S2) and showed close qualitative similarities to the dataset produced through random sampling in both models: pairwise correlations in channel expression are highly variable between channel pairs, with a positive correlations dominating but negative correlations also being found, while the first PC aligns with homogeneous scaling and the second PC has highly variable slopes in the different conductance planes (Fig. 5A).

This dataset is easily seen to be generated by two subspaces in the maximal conductance space (Fig. 5B): one characterized by variability solely in $g_{\mathrm{leak}}$ (triangles in Fig. 5B) and the other exhibiting variability exclusively in voltage-gated conductance ratios along DIC zero-sensitivity directions (crosses in Fig. 5B). Variability in $g_{\mathrm{leak}}$ only creates a degenerate dataset with strong, strictly positive correlations between conductance pairs, which isolates the effect of homogeneous scaling in channel conductances. Regression slopes of these subsets strongly align with the PC1 of the randomly sampled dataset. Variability limited to voltage-gated conductances (and fixed $g_{\mathrm{leak}}$) creates a degenerate dataset that also shows strong pairwise correlations. However, these correlations can be either positive or negative, and their regression slopes do not intersect the origin. Within this subset, the correlation between pairs of conductances arises from their distinct roles in shaping DIC values at threshold, and the slow DIC in particular. Channels that have an opposite effect on the slow DIC show a positive correlation (${\bar{g}}_{\mathrm{CaS}}$ and ${\bar{g}}_{A}$ in the STG), whereas channels that have similar effects show a negative correlation (${\bar{g}}_{\mathrm{CaL}}$ and ${\bar{g}}_{\mathrm{CaN}}$ in the DA model). The regression slopes within this subset strongly align with the PC2 of the complete dataset (compare PC2 in Fig. 5A with crosses in Fig. 5B).

This alternative approach to building degenerate parameter datasets shows that variable pairwise correlations in channel conductances could result from the interaction of two distinct factors: homogeneous scaling, which maintains the ratio between ion channel conductances; and degenerate conductance ratio, which leads to similar DIC values and hence similar membrane dynamical properties.

### Variability from both homogeneous scaling and degenerate conductance ratios blurs the connection between conductance correlation and function

**Fig. 6.**
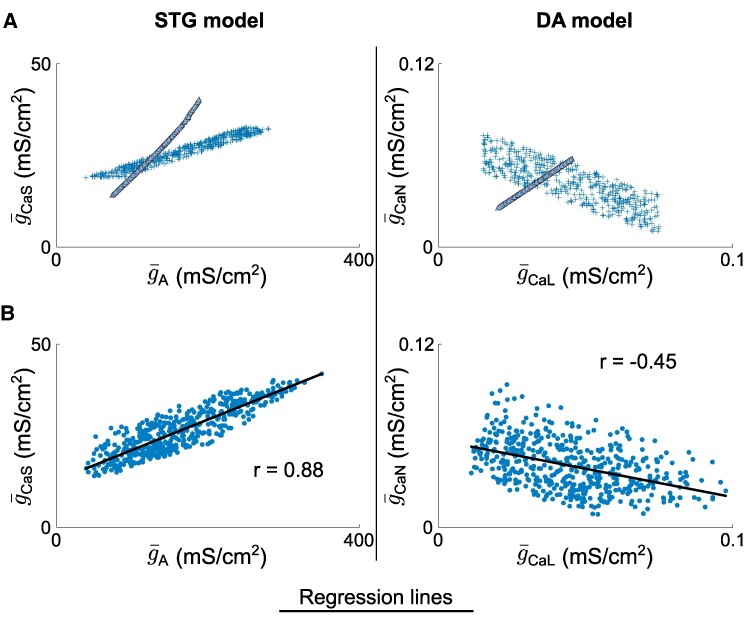
Variability from both homogeneous scaling and degenerate conductance ratios blurs the connection between conductance correlation and function. A) Scatter plots of custom generated populations separated into triangles (homogeneous scaling only) and crosses (variability in conductance ratios only) in the $\left({\bar{g}}_{A},{\bar{g}}_{\mathrm{CaL}}\right)$ 2D subspace for the STG model (left) and the $\left({\bar{g}}_{\mathrm{CaL}},{\bar{g}}_{\mathrm{CaN}}\right)$ 2D subspace for the DA model (right). B) Scatter plots of the full variability custom generated populations in the same 2D subspace as in a) for both the STG model (left) and the DA model (right), along with regression lines and Pearson correlation coefficients.

Our analysis so far shows that variability from homogeneous scaling creates strong positive correlations in channel conductances. Meanwhile, variability in voltage-gated conductance ratios also leads to strong correlations in channel conductances, but these can be either positive or negative depending on the channel pair (Fig. 6A). When both types of variability are present within a neuron population, these two correlation mechanisms interfere with each other to create highly variable levels of correlations between channel pairs (Fig. 6B). If both types of variability create positive correlations, the interference is minimal, and the global correlation in channel conductance remains strong (Fig. 6, left). However, if the variability in conductance ratios creates a negative correlation, the interference is consequential, and the global correlation in channel conductance becomes weak (Fig. 6, right). This observation is of interest, as it shows that the variable pairwise correlation observed in channel conductance values originate from potentially competing effects rather than from an actual uncorrelated role in our datasets.

From an experimental perspective, this analysis helps us to understand how recorded ion channel conductances are correlated. Homogeneous scaling always results in a strong positive correlation. Therefore, an overall positive correlation would indicate that the channels are functionally antagonistic, as their variable conductance ratios align with the direction of homogeneous scaling. Conversely, if the overall correlation is nonsignificant or slightly negative, this suggests that the channels are either functionally uncorrelated or agonistic. In these cases, the positive correlation from homogeneous scaling is counteracted by variability in conductance ratios, leading to a null or negative correlation, respectively. Experimentally, the normalization of channel conductances by input resistance can reveal correlations arising solely from variable conductance ratios, thus dissociating the two sources of degeneracy (see additional material for other 2D subspaces).

### Variability in pairwise correlations in conductance values is neuromodulation-dependent

**Fig. 7.**
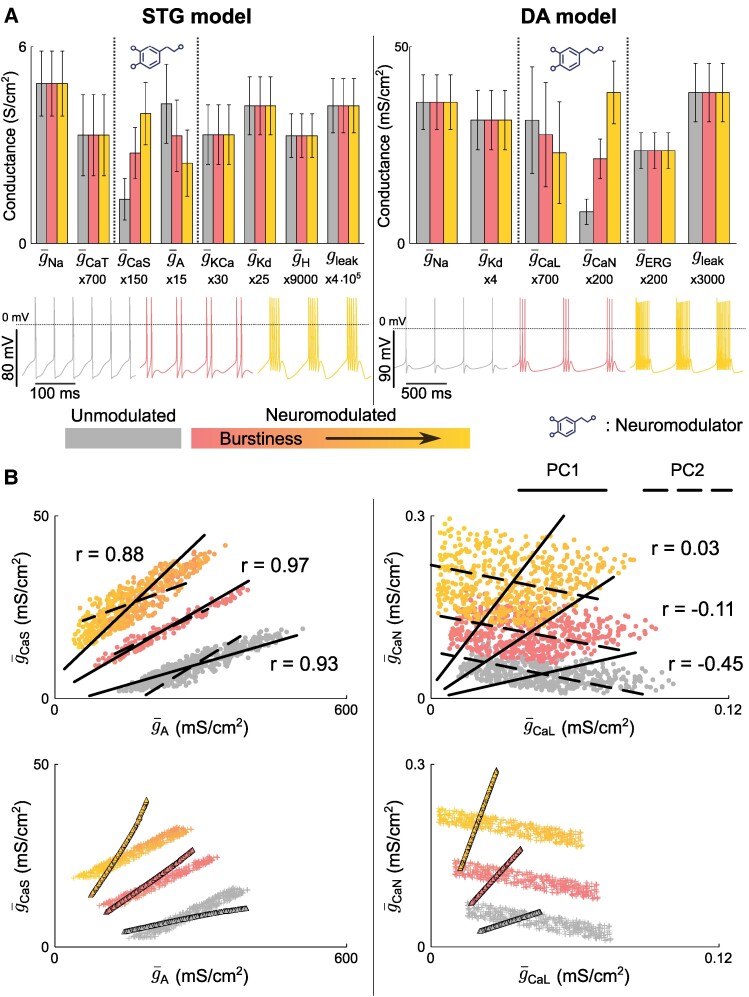
Variability in pairwise correlations in conductance values is neuromodulation-dependent. A) Bar plot of conductance values for custom generated populations in the three phenotypes considered for the STG model (right) and the DA model (left). The dotted line on the voltage traces corresponds to 0mV. B) Scatter plots of full variability custom generated populations in the neuromodulated 2D space for both the STG model (left) and the DA model (right) across three neuromodulated states, along with PC1, PC2, and Pearson correlation coefficients (top). Scatter plots of separated custom generated populations in the neuromodulated 2D space for both the STG model (left) and the DA model (right) across three neuromodulated states (bottom).

The variability in channel pairwise correlation level is therefore linked to the relative slope of the correlations created by both variability types, homogeneous scaling and degenerate conductance ratios. This has an interesting consequence when one studies the effect of neuromodulation on the correlation in channel conductance. To illustrate this consequence, we performed a simple computational experiment where we neuromodulated the excitability state of both models from spiking to light bursting to strong bursting (Fig. 7). In both cases, the neuromodulator affects the maximal conductance of two channel types: ${\bar{g}}_{A}$ and ${\bar{g}}_{\mathrm{CaS}}$ in the STG model, and ${\bar{g}}_{\mathrm{CaL}}$ and ${\bar{g}}_{\mathrm{CaN}}$ in the DA model (Fig. 7A). Those conductances are known to affect the burstiness of the respective neuron models. To create robust neuromodulation in degenerate neurons, we modulated the datasets of Fig. 5A by modifying the target threshold value for the slow DIC and used the algorithm of (7) to compute the neuromodulated conductance values for each neuron of the dataset (see Materials and methods). The resulting data points are shown in the scatter plots at the top of Fig. 7B. The dot color quantifies neuron burstiness, showing that the three firing patterns are robustly attained and well separated.

In both models, neuromodulation of neuron excitability strongly affects the level of pairwise correlations (Fig. 7B). In the STG model, the correlation between ${\bar{g}}_{A}$ and ${\bar{g}}_{\mathrm{CaS}}$ is strongly positive in spiking (*r* = 0.93), peaks in light bursting (*r* = 0.97), and decreases in strong bursting (*r* = 0.88). Meanwhile, in the DA model, the correlation between ${\bar{g}}_{\mathrm{CaL}}$ and ${\bar{g}}_{\mathrm{CaN}}$ is negative in spiking (r=−0.45), becomes less negative in light bursting (*r* = -0.11), and the two conductances appear uncorrelated in strong bursting (r=0.03). Pairwise correlations in ion channel conductances therefore appear neuromodulation-dependent.

The origin of these neuromodulation-dependent changes in pairwise correlations can be explained by plotting the first two principal components (PC1 and PC2) on the scatter plots and observing the effect of neuromodulation on them. On the one hand, neuromodulation creates a rotation of PC1 around the origin, which affects its slope. In the projections of Fig. 7B, the slope of PC1 increases when neurons switch from spiking to bursting in both models. This effect is consistent with the results obtained from homeostatic models of ion channel expression (14). On the other hand, neuromodulation creates a translation of PC2, and the slope is barely affected. As a result, the relative slopes between PC1 and PC2 depend on neuron neuromodulatory state, which affects the global correlation level.

In the STG model, both PC components have a positive slope. In spiking, PC1 has a flatter slope than PC2, which slightly widens the data cloud. As the model switches to bursting mode, the slope of PC1 increases and the two slopes become almost identical in light bursting. In this state, the two PCs align, which creates a strong correlation between the channel pair. As the model further increases its burstiness, the steepness of the slope of PC1 increases further and it becomes steeper than that of PC2. The two PCs disalign again and the correlation between the channel pair decreases. A similar observation can be made in the DA model, except that here PC2 has a negative slope. As a result, PC1 and PC2 become more and more orthogonal as burstiness increases, which reduces the correlation level, and even destroys the channel pairwise correlation in a strong bursting state.

As identified above, PC1 relates to the homogeneous scaling of conductances, whereas PC2 relates to the variability in the ratio between voltage-dependent conductances. To further demonstrate this link, we reproduced the three neuromodulatory states in two subsets where we isolated variability derived from homogeneous scaling (triangles in the bottom panels of Fig. 7B) from variability in conductance ratios (crosses in the bottom panels of Fig. 7B). We used the same algorithm as for the full dataset to create robustly neuromodulated states. The results from both models clearly show that robust neuromodulation is achieved through a rotation of the data points in the conductance space if variability derives from homogeneous scaling, whereas it is achieved through a translation of the data points if variability involves the ratio between voltage-dependent conductances.

This observation can be interpreted physiologically and provides significant insights into the requirements for robust neuromodulation in variable neurons. If robust neuromodulation is achieved through a rotation in the conductance space, it means that the robust neuromodulation rule is multiplicative: ${\bar{g}}_{i,\mathrm{MOD}}=\alpha_{i}\cdot {\bar{g}}_{i,\mathrm{init}}$ where $\alpha_{i}$ is set by the concentration of neuromodulator. The rule is multiplicative in the case of variability through homogeneous scaling, because neurons having twice the maximal conductance values require twice the change in conductance to achieve a similar firing pattern, owing to the change in input resistance. If robust neuromodulation is achieved through a translation in the conductance space, it means that the robust neuromodulation rule is additive: ${\bar{g}}_{i,\mathrm{MOD}}={\bar{g}}_{i,\mathrm{init}}+\beta_{i}$ where $\beta_{i}$ is also set by the neuromodulator concentration. The rule is additive in the case of variability in conductance ratios only because a similar firing pattern is achieved through a similar change in the normalized DIC values, which is created by the same change in maximal conductances. As a result, robust neuromodulation can be achieved through a simple, direct rule if only one type of variability is present in the neuronal population. However, derivation of a direct rule is impossible if both variability types are present in the population, which is likely considering the physiological significance of both types. Such a rule would indeed need to be both additive and multiplicative with a neuron-dependent ratio between both effects. Robust neuromodulation therefore requires an indirect rule involving a second messenger in highly degenerate neurons, which is precisely the mechanism observed in G protein-coupled receptor signaling.

### A simple indirect rule for robust neuromodulation in highly degenerate neurons

**Fig. 8.**
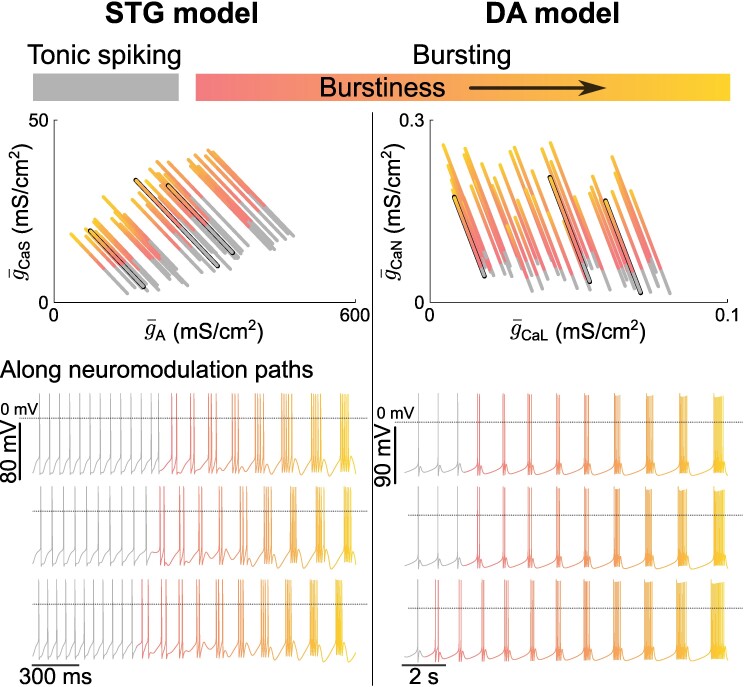
A simple indirect rule for robust neuromodulation in highly degenerate neurons. Neuromodulatory paths of custom generated populations for a gradually continuous neuromodulation application in the neuromodulated 2D subspace (top). Each line corresponds to one neuron continuously undergoing neuromodulation. Three randomly chosen neuron voltage traces along their neuromodulation paths (bottom). The dotted line on the voltage traces corresponds to 0mV.

We showed that robust neuromodulation in highly degenerate cells cannot rely on a simple rule directly targeting ion channels but rather requires a more complex rule involving a second messenger. This raises the questions of how complex a rule for reliable neuromodulation should be, and whether a general, model-independent rule could be derived. In an attempt to answer these questions, we used the algorithm developed above to construct reliable neuromodulatory paths in degenerate neurons for both STG and DA models, moving from tonic spiking to bursting of increasing burstiness (Fig. 8). Similar to the case presented above, the neuromodulatory algorithm targeted ${\bar{g}}_{A}$ and ${\bar{g}}_{\mathrm{CaS}}$ in the STG model, and ${\bar{g}}_{\mathrm{CaL}}$ and ${\bar{g}}_{\mathrm{CaN}}$ in the DA model. Many reliable neuromodulatory paths could be achieved in both models using a simple rule whose objective is to increase the target threshold value for the slow DIC while moving from tonic spiking to bursting, while keeping the ultraslow DIC value constant to maintain spiking and bursting periods (7) (see Materials and methods). Figure 8 plots the neuromodulatory pathways in the $\left({\bar{g}}_{\mathrm{CaS}},{\bar{g}}_{A}\right)$ plane (resp. $\left({\bar{g}}_{\mathrm{CaL}},{\bar{g}}_{\mathrm{CaN}}\right)$ plane) for the STG model (resp. DA model) and examples of neuromodulated neuronal traces.

Interestingly, although a simple direct rule cannot be used, the indirect rule resulted in linear neuromodulatory paths for both models, where the direction of neuromodulation is constant and only varies between neurons of different types. The nonlinearity occurs in the distance the neuron has to move in the direction to switch activity, which is affected by parameter variability (see the variability in the color transitions of Fig. 8 top). These results highlight that, even in the case of maximal degeneracy in neuron parameters, the relative change in maximal conductances of ion channels targeted by the same neuromodulatory receptor can be hard wired in a neuron type, creating a robust neuromodulatory path. The second messenger then has the role of controlling the movement along that neuromodulatory path that would lead to the target activity, strongly reducing the complexity of the reliable neuromodulation process. Such control could for example be implemented by sensing neuronal activity through intracellular calcium oscillations, as already suggested in homeostatic models (14, 25), or by sensing membrane voltage (23), creating activity-dependent changes in targeted maximal conductances. Substantial evidence of such activity-dependent neuromodulatory mechanisms involving intracellular calcium can be found in the literature on experimental studies (31–34).

## Discussion

### Two physiologically relevant sources of neuronal variability govern ion channel degeneracy

To uncover how so many different neuron types emerge, as well as the mechanisms underlying neuromodulation and variable neuronal response to neuroactive drugs, it is critical to understand how ion channels shape neuronal excitability (8, 13, 15, 17, 18). However, the connection between ion channels and neuronal signaling is complex due to channel degeneracy, and despite considerable advances made on the subject through experimental, computational, and mathematical work, a mechanistic understanding of ion channel variability and degeneracy in neurons remains elusive (2–6). Here, we showed that neuronal variability can be separated into two quantifiable, physiological components: homogeneous scaling of conductances and variability in conductance ratios.

Homogeneous scaling refers to the fact that neurons can exhibit similar activity if the relative difference in their channel maximal conductances is similar for all channels expressed at the membrane, whereby conductance ratios are maintained. This property has been observed experimentally in channel expression and shown to emerge from homeostatic rules (14, 27, 28). In this case, intrinsic characteristics are maintained, but responsiveness to synaptic input is altered due to differences in neuron input resistance. Variability in conductance ratios refers to the fact that neurons having a similar input resistance can exhibit similar activity with different ratios in their voltage-gated conductances. In this case, intrinsic characteristics are maintained, but the response to perturbations such as in temperature as well as channel blockade is altered due to differences in the relative role of each channel subtype on excitability.

Both sources of channel variability are physiologically relevant. Homogeneous scaling is central for network homeostasis, as it permits the tuning of neuron input/output response while keeping the intrinsic properties of neurons stable (35). Homogeneous scaling also permits compensation for changes in membrane capacitance. On the other hand, variability in conductance ratios permits improvement of the robustness against external perturbations by creating an heterogeneous response to perturbations affecting specific channel functions at the network level (36). It could also lead to variable inter-individual responses to neuroactive drugs.

The contributions of variability from homogeneous scaling and conductance ratios are intertwined in any neuron degenerate dataset, making any attempt at quantification difficult. Combining dimensionality reduction analysis and recent insights into the reduced dynamics of conductance-based models, we were able to separate the contributions of the two sources of variability, allowing the establishment of a mechanistic understanding of how variable ion channels can lead to specific neuronal activity. This enabled an understanding of the origin of ion channel variable pairwise correlations and the derivation of a robust indirect rule for reliable neuromodulation in degenerate neurons.

### Variable channel correlations arise from the interference between homogeneous scaling and variability in conductance ratios

Separating the effects of homogeneous scaling and variability in conductance ratios allowed analysis of the roles of the two sources of variability on channel pairwise correlations. Homogeneous scaling creates strictly positive correlations between all ion channels, and different firing patterns/neuron subtypes lead to different regression slopes, as observed in the channel expression data and homeostatic models of neuronal excitability (14). These positive correlations come from the passive role of ion channels on membrane properties through Ohm’s law: an increase of any channel conductance decreases the membrane input resistance. Other channels thus have to increase their conductance to maintain their effect on membrane potential variations.

On the other hand, variability in conductance ratios creates both positive and negative correlations between ion channel subsets, but not all ion channels. Ion channels correlate to maintain neuronal dynamics if their gating, representing either activation or inactivation, occurs on a similar timescale. The sign of the correlation is determined by the relative feedback provided by each channel gating on membrane potential variations, which is a key determinant of neuron dynamical properties as quantified by dynamic input conductances, for example (7). Specifically, activation of inward current and inactivation of outward current produce positive feedback, whereas activation of outward current and inactivation of inward current produce negative feedback. Two channels producing opposite feedbacks on a similar timescale will correlate positively (such as e.g. ${\bar{g}}_{A}$ and ${\bar{g}}_{\mathrm{CaS}}$ in the STG model), whereas two channels producing similar feedbacks will correlate negatively (such as e.g. ${\bar{g}}_{\mathrm{CaL}}$ and ${\bar{g}}_{\mathrm{CaN}}$ in the DA model).

When both sources of variability are present in a neuron degenerate set, the two types of correlations interfere with each other. When the correlation emerging from variability in conductance ratio is positive, both regression lines have a positive slope, creating an overall positive correlation whose intensity depends on the alignment of the regression lines. However, when the correlation emerging from variability in conductance ratio is negative, both regression lines have opposite signs, which can lead to an uncorrelation between two conductances even though there is a strong correlation between their role in neuron dynamics and passive properties. This situation could be indistinguishable from two channels that actually do not correlate due to a lack of action on a similar timescale. Therefore, variable correlations in channel conductances in a degenerate dataset do not always relate to correlated or uncorrelated functions but could also arise from highly correlated functions of opposite signs.

### The importance of indirect neuromodulatory pathways for reliable neuromodulation in variable neurons

One prominent issue arising from channel degeneracy involves how neuromodulation could be reliable across neurons when it acts on degenerate conductances (15–18, 32, 37). We showed that a simple direct rule for reliable neuromodulation could be derived if either homogeneous scaling or variability in conductance ratios, but not both, was present in a dataset. Indeed, homogeneous scaling requires a simple multiplicative rule due to its effect on input resistance, whereas variability in conductance ratios requires an additive rule. There is no direct rule if both variability types exist, as it would need to be both additive and multiplicative with a neuron-dependent ratio between the two effects.

We showed that a simple indirect rule could produce reliable neuromodulation when both sources of variability are present in the dataset. This rule is indirect in the sense that it uses an intermediate signaling pathway to connect neuromodulation concentration with changes in channel conductances. In our computational study, this intermediate pathway encodes the values of the slow and ultraslow dynamic input conductances around the threshold potential: neuromodulator concentration tunes the target values for both dynamic conductances, and a subset of ion channels are in turn modulated to reach these new targets. The presence of an intermediate messaging pathway is a core property of GPCR signaling, making such an indirect rule physiologically plausible. Our work provides a quantitative framework that provides a new angle of attack to study how intermediate signaling pathways could lead to reliable neuromodulation in degenerate neurons.

## Materials and methods

### Programming language

The Julia programming language was used in this work (38). Numerical integration was realized using *DifferentialEquations.jl*. Regression lines and correlations were computed using *Statistics.jl*. PCA was conducted using *LinearAlgebra.jl*.

### Conductance-based models

For all experiments, single-compartment conductance-based models were employed. These models articulate an ordinary differential equation for the membrane voltage *V*, where *N* ion channels are characterized as nonlinear dynamic conductances, and the phospholipid bilayer is represented as a passive resistor-capacitance circuit. Mathematically, the voltage–current relationship of any conductance-based neuron model is expressed as follows:


$$\begin{array}{rl} I_{C} & =C\frac{dV}{dt}+g_{\mathrm{leak}}(V-E_{\mathrm{leak}})=-I_{\mathrm{int}}+I_{\mathrm{ext}}\\ & =-\sum_{\mathrm{ion}\in I}g_{\mathrm{ion}}(V,t)(V-E_{\mathrm{ion}})+I_{\mathrm{ext}}. \end{array}$$

Here, *C* represents the membrane capacitance, $g_{\mathrm{ion}}$ denotes the considered ion channel conductance and is non-negative, gated between 0 (all channels closed) and ${\bar{g}}_{\mathrm{ion}}$ (all channels open), $E_{\mathrm{ion}}$ and $E_{\mathrm{leak}}$ are the channel reversal potentials, I is the index set of intrinsic ionic currents considered in the model, and $I_{\mathrm{ext}}$ is the current externally applied in vitro, or the combination of synaptic currents. Each ion channel conductance is nonlinear and dynamic, represented by $g_{\mathrm{ion}}(V,t)={\bar{g}}_{\mathrm{ion}}m_{\mathrm{ion}}^{a}(V,t)h_{\mathrm{ion}}^{b}(V,t)$, where $m_{\mathrm{ion}}$ and $h_{\mathrm{ion}}$ are variables gated between 0 and 1, modeling the opening and closing gates of ion channels, respectively. Throughout this study, both the isolated crab STG neuron model (25) and the adapted DA neuron model (26) (where SK channels had been blocked to enable bursting) were employed.

The STG model consists of seven ion channels that operate on various time scales: fast sodium channels (${\bar{g}}_{\mathrm{Na}}$); delayed-rectifier potassium channels (${\bar{g}}_{\mathrm{Kd}}$); T-type calcium channels (${\bar{g}}_{\mathrm{CaT}}$); A-type potassium channels (${\bar{g}}_{A}$); slow calcium channels (${\bar{g}}_{\mathrm{CaS}}$); calcium controlled potassium channels (${\bar{g}}_{\mathrm{KCa}}$); and H channels (${\bar{g}}_{H}$).

The DA model consists of six ion channels that operate on various time scales: fast sodium channels (${\bar{g}}_{\mathrm{Na}}$); delayed-rectifier potassium channels (${\bar{g}}_{\mathrm{Kd}}$); L-type calcium channels (${\bar{g}}_{\mathrm{CaL}}$); N-type calcium channels (${\bar{g}}_{\mathrm{CaN}}$); ERG channels (${\bar{g}}_{\mathrm{ERG}}$); and NMDA channels (${\bar{g}}_{\mathrm{NMDA}}$). Note that, owing to the multicellular nature of NMDA channels, they were excluded from this study but were still used for simulations with baseline values.

#### Random sampling sets

Random sampling sets consist of 200 neuron models with varying maximum ion channel conductances. These sets were created by generating numerous random points in the space of maximum ion channel conductances (within specified ranges). Subsequently, the models underwent post-processing based on their firing patterns, with only those fitting the desired phenotype being retained. For the STG models, post-processing involved considerations of peak and hyperpolarized voltages, intra- and interburst frequencies, the number of spikes per burst, and burstiness (computed as in Ref. (39)). Meanwhile, the DA models were post-processed based on their peak and hyperpolarized voltages and spike frequency.

#### Dynamic input conductances

DICs consist of three voltage-dependent conductances that separate according to timescales: one fast, one slow, and one ultraslow, denoted as $g_{f}(V)$, $g_{s}(V)$, and $g_{u}(V)$, which can be computed as linear functions of the maximal conductance vector ${\bar{g}}_{\mathrm{ion}}\in R^{N}$ of an *N*-channel conductance-based model at each voltage level *V*:


$$[g_{f}(V);g_{s}(V);g_{u}(V)]=f_{\mathrm{DIC}}(V)=S(V)\cdot {\bar{g}}_{\mathrm{ion}}\;,$$

where $S(V)\in R^{3\times N}$ is a sensitivity matrix that can be built by: $S_{i\;j}(V)=-(w_{i\;j}\cdot \frac{\partial \dot{V}}{\partial X_{j}}\frac{\partial X_{\;j,\infty }}{\partial V})/g_{\mathrm{leak}}$, where *i* denotes the timescale, $X_{j}$ are gating variables of the *j*th channel of the considered model and $w_{i\;j}$ is a timescale-dependent weight which is computed as the logarithmic distance of the time constant of $X_{j}$ and the timescale *i* (7). While the complete curve of the DICs may be of interest, only its value at the threshold voltage $V_{\mathrm{th}}$ is used, as the values and signs of the DICs at $V_{\mathrm{th}}$ reliably determine the firing pattern (7). Thus, the following linear system $f_{\mathrm{DIC}}(V_{\mathrm{th}})=S(V_{\mathrm{th}})\cdot {\bar{g}}_{\mathrm{ion}}$ makes the link between ion channel conductances and neuronal activity.

#### An efficient method to build sets that allow the separation of the two sources of degeneracy

Throughout this study, a novel method for generating degenerate datasets of conductance-based models has been developed, which was proven to be significantly faster than the random sampling approach (all figures were created using a dataset of 500 neurons). The methodology for a *N*-channel conductance-based model can be summarized as follows:

1. The leakage conductance $g_{\mathrm{leak}}$ is drawn from a physiological uniform distribution: $g_{\mathrm{leak}}∼U(g_{\mathrm{leak}\;\;\min},g_{\mathrm{leak}\;\;\max})$;
2.
N−3 maximum ion channel conductances are drawn from a physiological uniform distribution that is proportional to $g_{\mathrm{leak}}$: ${\bar{g}}_{\mathrm{ion}}∼\frac{g_{\mathrm{leak}}}{(g_{\mathrm{leak}\;\;\min}+g_{\mathrm{leak}\;\;\max})/2}\cdot U^{N-3}({\bar{g}}_{\mathrm{unmod}\;\min},{\bar{g}}_{\mathrm{unmod}\;\max})$;
3. The three remaining maximum ion channel conductances are computed using the linear system $f_{\mathrm{DIC}}(V_{\mathrm{th}})=S(V_{\mathrm{th}})\cdot {\bar{g}}_{\mathrm{ion}}$, in which $f_{\mathrm{DIC}}(V_{\mathrm{th}})$ are fixed by the user to choose the firing pattern of the population.

The normalization by $g_{\mathrm{leak}}$ in (2) allows the combination of the effects of homogeneous scaling and variability in conductance ratios. The subsequent sets, each targeting either homogeneous scaling or conductance ratio, were generated by using shared deterministic values for $g_{\mathrm{leak}}$ or for the N−3 maximum ion channel conductances, respectively. The zero-sensitivity directions of slow dynamical membrane properties were computed using the equations for the slow dynamic input conductance, where the two ion channel conductances of interest were treated as variables along this direction.

#### Neuromodulation algorithm

As a result of this newly developed method for generating degenerate neuronal sets, neuromodulation of these sets is achieved by manipulating the linear system $f_{\mathrm{DIC}}(V_{\mathrm{th}})=S(V_{\mathrm{th}})\cdot {\bar{g}}_{\mathrm{ion}}$. Once a population is created, the values of $f_{\mathrm{DIC}}(V_{\mathrm{th}})$ can be adjusted, and the linear system can be solved for certain ion channel conductances (the modulated ones) to achieve a new firing pattern. Specifically, two maximum conductances are recalculated by tuning the value of the slow dynamic input conductance while the ultraslow dynamic input conductance is kept unchanged. The latest results were obtained by continuously adjusting this slow dynamic input conductance value.

## Supplementary Material

pgae415_Supplementary_Data

## Data Availability

All code and data can be found on the first author’s GitHub (https://github.com/arthur-fyon/CORR_2024) (40).

## Preprints

This manuscript was posted on a preprint: arXiv:2405.02038.
