# Supplementary material for: Dimensionality reduction of neuronal degeneracy reveals two interfering physiological mechanisms
Source: PNAS Nexus. 2024 Sep 19;3(10):pgae415. doi: 10.1093/pnasnexus/pgae415 (PMC11443964; doi:10.1093/pnasnexus/pgae415)
Supplement: pgae415_Supplementary_Data [file pgae415_supplementary_data.pdf]

Supplemental methods: Dimensionality reduction of neuronal degeneracy reveals two interfering physiological mechanisms

Code and data can be found in the first author GitHub (https://github.com/arthur-fyon/CORR_2024).

Simulation details for the two conductance based models

Throughout the entire paper, two high-dimensional conductance-based models have been employed. First, the voltage equation for the Stomatogastric Ganglion (STG) neuron model, as proposed by Liu et al. [S1], is expressed as follows:

$$\begin{aligned} C\dot{V} = &- \bar{g}_{\text{Na}} m_{\text{Na}}^3 h_{\text{Na}} (V - E_{\text{Na}}) - \bar{g}_{\text{CaT}} m_{\text{CaT}}^3 h_{\text{CaT}} (V - E_{\text{Ca}}) - \bar{g}_{\text{CaS}} m_{\text{CaS}}^3 h_{\text{CaS}} (V - E_{\text{Ca}}) \\ &- \bar{g}_{\text{A}} m_{\text{A}}^3 h_{\text{A}} (V - E_{\text{K}}) - \bar{g}_{\text{KCa}} m_{\text{KCa}}^4 (V - E_{\text{K}}) - \bar{g}_{\text{Kd}} m_{\text{Kd}}^4 (V - E_{\text{K}}) \\ &- \bar{g}_{\text{H}} m_{\text{H}} (V - E_{\text{H}}) - g_{\text{leak}} (V - E_{\text{leak}}) + I_{\text{app}}, \end{aligned} \tag{1}$$

where the dot notation represents the time derivative, C is the membrane capacitance, Na stands for the sodium current, CaT for the T-type calcium current, CaS for the slow calcium current, A for the A-type potassium current, KCa for the calcium-controlled potassium current, Kd for the delayed rectified potassium current, H for the H current, and leak for the leakage current. All E values denote the channel reversal Nernst potentials, and I_{app} represents the externally applied current. The parameters of interest are the maximum ion channel conductances $\bar{g}$, expressed in mS/cm^2, corresponding to the ion channel conductance when fully opened. Additionally, all m and h variables represent activation and inactivation gate variables, respectively, following first-order lag equations that are voltage-dependent. Notably, the KCa gating variable also depends on intracellular calcium concentration, and its ordinary differential equation (ODE) can be found in Liu et al. [S1]. For all STG voltage traces in this article, equation (1) and subsequent gate variable equations are numerically integrated using the Julia language [S2].

Second, the voltage equation for the Dopaminergic (DA) neuron model, adapted from Qian et al. [S3] (with the SK channels blocked), is given by:

$$\begin{aligned} C\dot{V} = &- \bar{g}_{\text{Na}} m_{\text{Na}}^3 h_{\text{Na}} (V - E_{\text{Na}}) - \bar{g}_{\text{Kd}} m_{\text{Kd}}^3 (V - E_{\text{K}}) - \bar{g}_{\text{CaL}} m_{\text{CaL}}^2 (V - E_{\text{Ca}}) \\ &- \bar{g}_{\text{CaN}} m_{\text{CaN}} (V - E_{\text{Ca}}) - \bar{g}_{\text{ERG}} m_{\text{ERG}} (V - E_{\text{K}}) - g_{\text{leak}} (V - E_{\text{leak}}) \\ &- \bar{g}_{\text{NMDA}} \frac{(V - E_{\text{NMDA}})}{1 + Mg \cdot \exp(-0.08V)/10} + I_{\text{app}}, \end{aligned} \tag{2}$$

where CaL represents the L-type calcium current, CaN the N-type calcium current, ERG the ERG current, and NMDA the NMDA current. Additionally, Mg denotes the extracellular magnesium concentration, assumed to be a constant of 1.4 µM. For all dopaminergic (DA) voltage traces in this article, equation (2) and subsequent gate variable equations are numerically integrated using the Julia language [S2]. It is important to note that the NMDA ion channel is not discussed in the article and is treated with a constant maximum conductance scaled by the leakage current.

Computation details for the Dynamic Input Conductances (DICs)

This article employs the concept of Dynamic Input Conductances (DICs) introduced in Drion et al. [S4]. DICs consist of three voltage-dependent conductances that separate according to timescales: one

fast, one slow, and one ultraslow. These DICs have been demonstrated to shape neuronal spiking. Specifically, based on specific values of the DICs, it becomes possible to predict the firing pattern of the neuron. The computation of the DICs in this article has been improved compared to Drion et al. [S4], and this enhancement will be detailed in the following sections.

The DICs are three voltage-dependent conductances, denoted as $g_\mathrm{f}(V)$, $g_\mathrm{s}(V)$, and $g_\mathrm{u}(V)$, which can be computed as linear functions of the maximal conductance vector $\bar{g}_\mathrm{ion} \in \mathbb{R}^N$ of an N-channel conductance-based model at each voltage level V

$$\begin{bmatrix} g_\mathrm{f}(V) \\ g_\mathrm{s}(V) \\ g_\mathrm{u}(V) \end{bmatrix} = f_\mathrm{DIC}(V) = S(V) \cdot \bar{g}_\mathrm{ion} \,, \tag{3}$$

where $S(V) \in \mathbb{R}^{3\times N}$ is a sensitivity matrix that can be built by (line per line)

$$S_{\mathrm{f}\,j}(V) = -\left(w_{\mathrm{fs},X_j} \cdot \frac{\partial \dot{V}}{\partial X_j} \frac{\partial X_{j,\infty}}{\partial V} \right) \Big/ g_\mathrm{leak}, \tag{4}$$

$$S_{\mathrm{s}\,j}(V) = -\left((w_{\mathrm{su},X_j} - w_{\mathrm{fs},X_j}) \cdot \frac{\partial \dot{V}}{\partial X_j} \frac{\partial X_{j,\infty}}{\partial V} \right) \Big/ g_\mathrm{leak}, \tag{5}$$

$$S_{\mathrm{u}\,j}(V) = -\left((1 - w_{\mathrm{su},X_j}) \cdot \frac{\partial \dot{V}}{\partial X_j} \frac{\partial X_{j,\infty}}{\partial V} \right) \Big/ g_\mathrm{leak}, \text{ with } j = 1:N, \tag{6}$$

where equations (4), (5), and (6) represent the computation of rows 1, 2, and 3, respectively, of the matrix $S(V)$ — that is, the computation of the fast, slow, and ultraslow DICs. The weighing factors w_{fs,X_j} and w_{su,X_j} are computed following the method outlined in Drion et al. [S4], where X_j corresponds to the gating variable(s) (activation and/or inactivation) of the current with index j, and $X_{j,\infty}$ denotes the steady-state function(s) of the considered gating variable lag equation(s). It is important to note that if the current j has both activation and inactivation variables, the column S_j corresponds to the sum of equations (4) through (6) over the two gating variables of the current j.

While the complete curve of the DICs may be of interest, only its value at the threshold voltage V_th is used, as the values and signs of the DICs at V_th reliably determine the neuronal firing pattern [S4]. In the following sections, the voltage dependency of all variables will be disregarded, as functions are only evaluated at V_th. The threshold voltage is computed as $g_\mathrm{in}(V_\mathrm{th}) = g_\mathrm{f}(V_\mathrm{th}) + g_\mathrm{s}(V_\mathrm{th}) + g_\mathrm{u}(V_\mathrm{th}) < 0$, while ensuring that $g_\mathrm{in}(V_\mathrm{th} - \delta V) \geq 0$ with any arbitrarily small $\delta V > 0$. It is important to note that this algorithm might fail for the DA neuron model, where the default $V_\mathrm{th} = -55.5$ mV.

Computation details for the random sampling sets (Figures 1, 2, 3 and 4)

For both models, random sampling sets consist of randomly drawing maximum ion channel conductances, *i.e.*, generating a point in the conductance space, numerically integrating the model equations, and retaining the point in the set if its phenotype is correct until the set reaches 200 neurons, according to different criteria. For the STG model, a bursting behavior is desired, so post-processing is carried out on:

- Peak voltage (between 49.9 mV and 49.95 mV);
- Low voltage (between -76 mV and -69 mV);
- Number of spike per burst (5);
- Burstiness (between 3000 Hz^2 and 7000 Hz^2);
- Interburst frequency (between 8.8 Hz and 9.9 Hz);
- Intraburst frequency (between 70 Hz and 140 Hz);

with the burstiness computed as in Franci et al. [S5].

For the DA model, a pacemaking tonic spiking behavior is desired, so post-processing is carried out on:

- Peak voltage (between 54 mV and 58.5 mV);
- Low voltage (between -80.5 mV and -77.5 mV);
- Spiking frequency (between 1.75 Hz and 1.95 Hz).

To generate a random point in the parameter space for each N-channel conductance-based model, each conductance was drawn from a uniform random distribution $\bar{g}_{\text{ion}} \sim \mathcal{U}^N(0, \bar{g}_{\text{ion max}})$ and $g_{\text{leak}} \sim \mathcal{U}(0, g_{\text{leak max}})$, with $\bar{g}_{\text{ion max}}$ and $g_{\text{leak max}}$ are equal to the corresponding values in Tables 1 and 2 for the STG and DA models, respectively. It is noteworthy that, for the DA model, the NMDA conductance is computed as $\bar{g}_{\text{NMDA}} = g_{\text{leak}} \cdot 0.12/0.013$, *i.e.*, a baseline value of 0.12 scaled by the leakage conductance to achieve homogeneous scaling.

Current name	Na	CaT	CaS	A	KCa	Kd	H	leak
$\bar{g}_{\text{ion max}}$ or $g_{\text{leak max}}$	8000	12	50	600	250	350	0.7	0.02

Table 1: Right boundary of the uniform distribution for all maximum ion channel conductances of the STG model.

Current name	Na	Kd	CaL	CaN	ERG	leak
$\bar{g}_{\text{ion max}}$ or $g_{\text{leak max}}$	60	20	0.1	0.12	0.25	0.02

Table 2: Right boundary of the uniform distribution for all maximum ion channel conductances of the DA model.

Additional 2D subspaces of the random sampling datasets normalized by the input resistance (Figure 4)

Fig. S1 depicts a scatter matrix of the random sampling datasets normalized by the input resistance for the 2D subspaces of Fig. 5 from the main manuscript. This visualization highlights that normalizing by the input resistance effectively removes the effect of homogeneous scaling, leaving only mechanistic correlations that might arise from the ion channels.

On one hand, antagonist channels reveal positive correlations once normalized (*e.g.*, see $\bar{g}_{\text{A}}$ and $\bar{g}_{\text{CaS}}$ for the STG model and $\bar{g}_{\text{Kd}}$ and $\bar{g}_{\text{CaL}}$ for the DA model). Such correlations within the variability of conductance ratios align with homogeneous scaling, leading to overall positive correlations in the random sampling datasets (see Fig. 1 of the main manuscript).

On the other hand, agonist channels reveal negative correlations once normalized (*e.g.*, $\bar{g}_{\text{CaL}}$ and $\bar{g}_{\text{CaN}}$ for the DA model). Such correlations within the variability of conductance ratios oppose homogeneous scaling, leading to overall negative or nonexistent correlations in the random sampling datasets, depending on the strength of homogeneous scaling (see Fig. 1 of the main manuscript).

A last case is when the channels are not correlated mechanistically, such as the pair including ultraslow channels like $\bar{g}_{\text{H}}$ for the STG model or $\bar{g}_{\text{ERG}}$ for the DA model. As the dynamics of such channels lie in another timescale than other channels, the correlation within the normalized dataset is nonexistent. When combined with homogeneous scaling, this may lead to either a nonexistent correlation or a slight positive correlation, depending on the strength of homogeneous scaling (see Fig. 1 of the main manuscript).

Note that input resistance have been computed as the sum of the ion channel conductances at -60 mV for the STG model and -55.5 mV for the DA model.

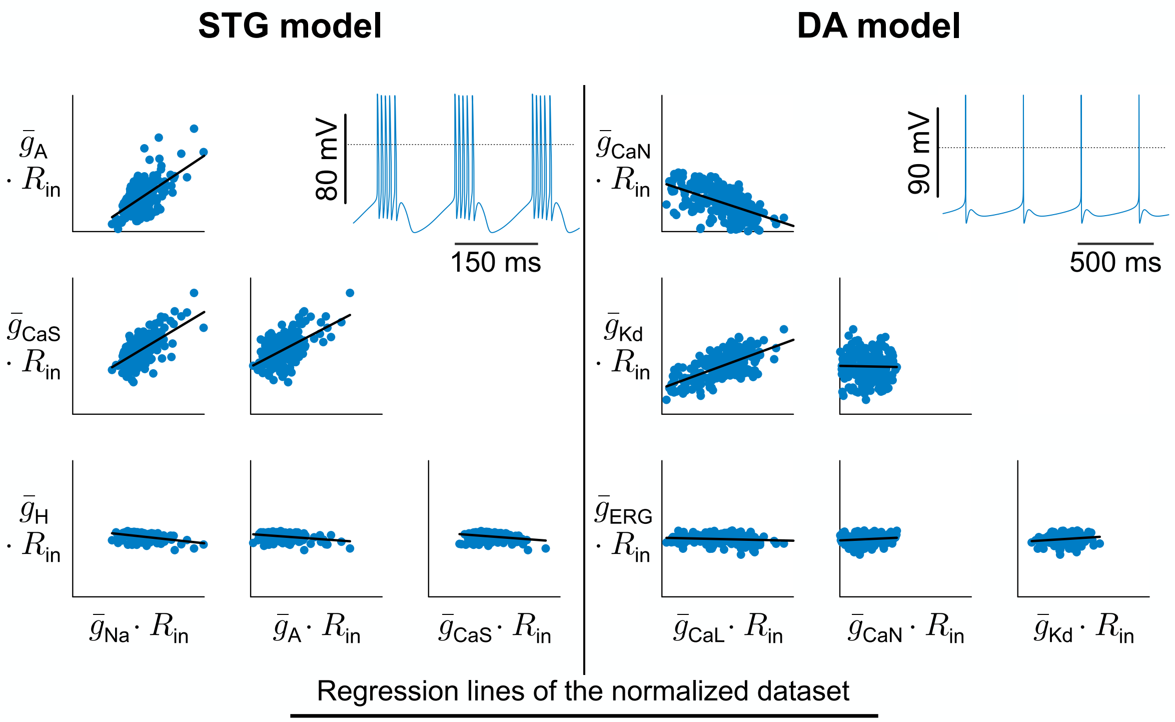

Fig. S1: Scatter matrices of random sampling populations in the conductance spaces normalized by the input resistance for the STG model (left) and the DA model (right) along with the directions of the regression lines. The 2D subspaces shown here do not represent all conductances of the models and are chosen to be the same as in Fig. 5 of the main manuscript. All normalized conductances are dimensionless. The bottom left corner of each 2D subspace represents the origin of the conductance space, and ranges are irrelevant. The dotted line on the voltage traces corresponds to the 0 mV line.

Computation details for the efficient method to build degeneracy sets that allows to remove the effect of homogeneous scaling (Figures 5, 6, 7 and 8)

Throughout this study, a novel method for generating degenerate datasets of conductance-based models has been developed, proving to be significantly faster than the random sampling approach (all figures were created using a dataset of 500 neurons). The methodology for an N-channel conductance-based model can be summarized as follows:

1. The leakage conductance g_{leak} is drawn from a physiological uniform distribution: $g_{\text{leak}} \sim \mathcal{U}\left(g_{\text{leak min}}, g_{\text{leak max}}\right)$;
2. N - 3 maximum ion channel conductances are drawn from a physiological uniform distribution that is proportional to g_{leak}: g_{leak}: $\bar{g}_{\text{ion}} \sim \frac{g_{\text{leak}}}{(g_{\text{leak min}}+g_{\text{leak max}})/2} \cdot \mathcal{U}^{N-3}\left(\bar{g}_{\text{unmod min}}, \bar{g}_{\text{unmod max}}\right)$
3. The 3 remaining maximum ion channel conductances are computed using the compensation algorithm described in Drion et al. [S4], and detailed just below.

So, the maximal conductance vector $\bar{g}_{\text{ion}} \in \mathbb{R}^N$ of a N-channel conductance-based model can be split in two parts: $\bar{g}_{\text{ion random}} \in \mathbb{R}^{N-3}$ that corresponds to the N - 3 randomly initialized maximum conductances in step 2 (proportional to g_{leak}), and $\bar{g}_{\text{ion compensated}} \in \mathbb{R}^3$ which corresponds to the 3 maximum conductances computed in step 3. The same split can be applied to S: $S_{\text{random}} \in \mathbb{R}^{3\times N-3}$

and $S_{\text{compensated}} \in \mathbb{R}^{3\times 3}$. By doing so, the DIC equation (3) can be written as:

$$f_{\text{DIC}} = S_{\text{random}} \cdot \bar{g}_{\text{ion random}} + S_{\text{compensated}} \cdot \bar{g}_{\text{ion compensated}}$$
$$\Longleftrightarrow S_{\text{compensated}} \cdot \bar{g}_{\text{ion compensated}} = f_{\text{DIC}} - S_{\text{random}} \cdot \bar{g}_{\text{ion random}}. \quad (7)$$

If values of the DIC at threshold voltage are specified, equation (7) consists of a system of 3 equations with 3 unknowns, *i.e.*, $\bar{g}_{\text{ion compensated}}$. For this system to be solved, $S_{\text{compensated}}$ must be of full rank. This means that the compensated ion channels must have a significant impact on the three timescales defined by the DICs. For the STG model, the compensated ones are the sodium (fast), A-type potassium (slow), and H (ultraslow) currents. For the DA model, the compensated ones are the sodium (fast), N-type calcium (slow), and ERG (ultraslow) currents.

So, the inputs of this algorithm consist of an a priori threshold voltage (which can be computed after using the algorithm described above), the values of the 3 DICs at the threshold voltage (these will specify the desired firing pattern), and all the boundaries of the uniform distributions. Note that, very often, a linear relation can be found between the value of the slow DIC at the threshold voltage and the corresponding value of the fast DIC. Tables 3, 4, and 5 contain all the parameters for the generated sets in the article. Also, note that the NMDA conductances follow the same formula as in random sampling sets.

	V_{th}	$g_{\text{f}}(V_{\text{th}})$	$g_{\text{s}}(V_{\text{th}})$	$g_{\text{u}}(V_{\text{th}})$
STG model	-50 mV	-$g_{\text{s}}(V_{\text{th}})$ - 2.2	-8	4
DA model	-55.5 mV	-3.9·$g_{\text{s}}(V_{\text{th}})$ - 11	0.5	5

Table 3: Inputs for the DIC set generation algorithm for both models.

Current name	CaT	CaS	KCa	Kd	leak
$\bar{g}_{\text{ion min}}$ or $g_{\text{leak min}}$	2	6	140	70	0.007
$\bar{g}_{\text{ion max}}$ or $g_{\text{leak max}}$	7	22	180	140	0.014

Table 4: Boundaries of the uniform distribution for all maximum ion channel conductances of the STG model.

Current name	Kd	CaL	leak
$\bar{g}_{\text{ion min}}$ or $g_{\text{leak min}}$	0.015	6	0.008667
$\bar{g}_{\text{ion max}}$ or $g_{\text{leak max}}$	0.075	10	0.017334

Table 5: Boundaries of the uniform distribution for all maximum ion channel conductances of the DA model.

Fig. S2 depicts the firing pattern characteristics, including inter- and intra-burst frequencies, duty cycle for the STG model, and spiking frequency for the DA model, at the population level for the sets generated by DICs. This visualization demonstrates the similarity of these neurons to those from the random sampling sets in terms of phenotype variability.

For the homogeneous scaling sets only, the boundaries $\bar{g}_{\text{ion random}}$ of the uniform distributions coincide to only keep the scaling with respect to g_{leak}, *i.e.*, $\bar{g}_{\text{ion min}} = \bar{g}_{\text{ion max}}$. Tables 6 and 7 contain these these boundaries for the homogeneous scaling-only generated sets in the article.

Current name	CaT	CaS	KCa	Kd
$\bar{g}_{\text{ion min}}$ or $\bar{g}_{\text{ion max}}$	3.5	11	160	110

Table 6: Boundaries of the uniform distribution for all maximum ion channel conductances of the STG model, for the homogeneous scaling only set.

The last sets generated in this article consist of those where full variability is retained, except for homogeneous scaling. This implies that only the variability in conductance ratios is preserved. To achieve this, the boundaries of the uniform distribution for the leakage conductance coincide, *i.e.*, $g_{\text{leak min}} = g_{\text{leak max}}$. Consequently, g_{leak} is a constant. For the STG model, $g_{\text{leak}} = 0.01$, while for the DA model, $g_{\text{leak}} = 0.013$.

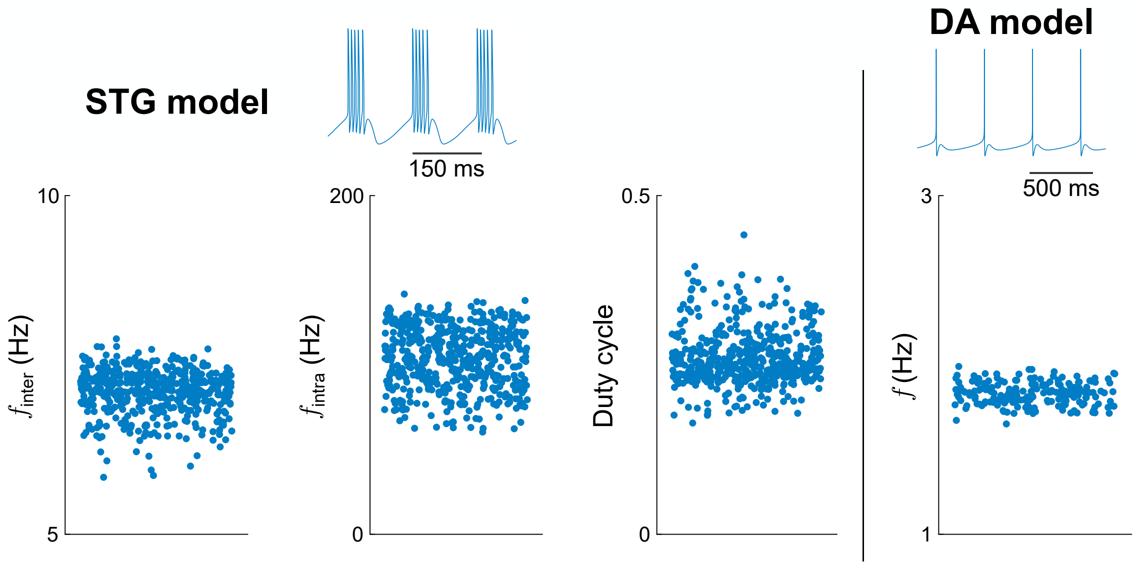

Fig. S2: Firing pattern characteristics for the full variability DICs generated sets for both model. On the left, the interburst frequencies, intraburst frequencies, and duty cycles of the DICs generated STG neurons are depicted. On the right, the spiking frequencies of the DICs generated DA neurons are depicted.

Current name	Kd	CaL
$\bar{g}_{\text{ion min}}$ or $\bar{g}_{\text{ion max}}$	0.03	6

Table 7: Boundaries of the uniform distribution for all maximum ion channel conductances of the DA model, for the homogeneous scaling only set.

Computation details for the neuromodulation algorithm (Figures 7 and 8)

A neuromodulation algorithm can be used to neuromodulate a degenerate set that has been initialized without the method previously described. This neuromodulation algorithm is greatly inspired by the set generation method described above.

Two conductances can be chosen to be neuromodulated in each model: $\bar{g}_{\text{CaS}}$ and $\bar{g}_{\text{A}}$ for the STG model, and $\bar{g}_{\text{CaL}}$ and $\bar{g}_{\text{CaN}}$ for the DA model. Moreover, neuromodulation is only applied to the slow and ultraslow DICs in this paper, as the transition from tonic spiking to bursting is studied. This means that the first row of S is dropped, resulting in a new matrix $S_{\text{su}} \in \mathbb{R}^{2\times N}$ corresponding to the second and third rows of S.

So a similar split can be made for the maximal conductance vector $\bar{g}_{\text{ion}} \in \mathbb{R}^N$ of an N-channel conductance-based model and its reduced sensitivity matrix S_{su}: $\bar{g}_{\text{ion unmod}} \in \mathbb{R}^{N-2}$ corresponds to the $N-2$ unmodulated (unchanged) maximum conductances and $\bar{g}_{\text{ion mod}} \in \mathbb{R}^2$ corresponds to the 2 maximum conductances that are neuromodulated, along with their corresponding columns in S_{su}. Additionally, $S_{\text{su unmod}} \in \mathbb{R}^{2\times N-2}$ and $S_{\text{su mod}} \in \mathbb{R}^{2\times 2}$. By doing so, the DIC equation (3) can be written as

$$\begin{bmatrix} g_{\text{s}} \\ g_{\text{u}} \end{bmatrix} = f_{\text{su DIC}} = S_{\text{su unmod}} \cdot \bar{g}_{\text{ion unmod}} + S_{\text{su mod}} \cdot \bar{g}_{\text{ion mod}}$$
$$\iff S_{\text{su mod}} \cdot \bar{g}_{\text{ion mod}} = f_{\text{su DIC}} - S_{\text{su unmod}} \cdot \bar{g}_{\text{ion unmod}}. \qquad (8)$$

If values of the slow and ultraslow DICs at the threshold voltage are specified, equation (8) consists of a system of 2 equations with 2 unknowns, *i.e.*, $\bar{g}_{\text{ion mod}}$. For this system to be solved, $S_{\text{su mod}}$ must be of full rank, meaning that the neuromodulated ion channels must have a significant impact on the two timescales defined by the DICs.

If the values of the slow and ultraslow DICs are the same as in the set generation mechanism, the computed neuromodulated conductances match the original ones. However, as soon as the DIC values are modified, the modulated conductances will be tuned to match the desired DIC values, *i.e.*, the desired firing pattern. In this article, we showed that neuromodulation (for the tonic spiking to bursting transition) is robust when the ultraslow DIC value is kept constant (the same as in the generation mechanism) and when the slow DIC value is tuned to achieve different firing patterns. Concerning the STG model, $g_\text{s} = 5$ for the tonic spiking set and $g_\text{s} = -2$ for the light bursting set. Regarding the DA model, $g_\text{s} = -1.5$ for the light bursting set and $g_\text{s} = -4$ for the strong bursting set.

For Figure 8, the same neuromodulation was used, but with continuous variation from one extreme value of g_s to another. Steps of 0.02 in g_s were used for the STG model, while steps of 0.005 in g_s were used for the DA model.

References

[S1] Zheng Liu, Jorge Golowasch, Eve Marder, and LF Abbott. A model neuron with activity-dependent conductances regulated by multiple calcium sensors. *Journal of Neuroscience*, 18(7):2309–2320, 1998.

[S2] Jeff Bezanson, Alan Edelman, Stefan Karpinski, and Viral B Shah. Julia: A fresh approach to numerical computing. *SIAM Review*, 59(1):65–98, 2017.

[S3] Kun Qian, Na Yu, Kristal R Tucker, Edwin S Levitan, and Carmen C Canavier. Mathematical analysis of depolarization block mediated by slow inactivation of fast sodium channels in midbrain dopamine neurons. *Journal of Neurophysiology*, 112(11):2779–2790, 2014.

[S4] Guillaume Drion, Alessio Franci, Julie Dethier, and Rodolphe Sepulchre. Dynamic input conductances shape neuronal spiking. *eNeuro*, 2(1), 2015.

[S5] Alessio Franci, Guillaume Drion, and Rodolphe Sepulchre. Robust and tunable bursting requires slow positive feedback. *Journal of Neurophysiology*, 119(3):1222–1234, 2018.
